# Position Weight Matrix, Gibbs Sampler, and the Associated Significance Tests in Motif Characterization and Prediction

**DOI:** 10.6064/2012/917540

**Published:** 2012-10-23

**Authors:** Xuhua Xia

**Affiliations:** Department of Biology, University of Ottawa, 30 Marie Curie, Ottawa, ON, Canada K1N 6N5

## Abstract

Position weight matrix (PWM) is not only one of the most widely used bioinformatic methods, but also a key component in more advanced computational algorithms (e.g., Gibbs sampler) for characterizing and discovering motifs in nucleotide or amino acid sequences. However, few generally applicable statistical tests are available for evaluating the significance of site patterns, PWM, and PWM scores (PWMS) of putative motifs. Statistical significance tests of the PWM output, that is, site-specific frequencies, PWM itself, and PWMS, are in disparate sources and have never been collected in a single paper, with the consequence that many implementations of PWM do not include any significance test. Here I review PWM-based methods used in motif characterization and prediction (including a detailed illustration of the Gibbs sampler for *de novo* motif discovery), present statistical and probabilistic rationales behind statistical significance tests relevant to PWM, and illustrate their application with real data. The multiple comparison problem associated with the test of site-specific frequencies is best handled by false discovery rate methods. The test of PWM, due to the use of pseudocounts, is best done by resampling methods. The test of individual PWMS for each sequence segment should be based on the extreme value distribution.

## 1. Introduction 

Most genetic switches are in the form of sequence motifs that interact with proteins [1]. Position weight matrix or PWM [2–6] is one of the key bioinformatic tools used extensively in characterizing and predicting motifs in nucleotide and amino acid sequences. The popularity of PWM has been further increased since its implementation as a component in PSI-BLAST [7], which is frequently used to generate PWM for motif characterization and prediction [8–11].

PWM has been applied extensively in studies of cis-regulatory elements in the genome such as translation initiation sites [12], transcription initiation sites [13], transcription factor binding sites [14–22], yeast intron splicing sites [23], whole-genome identification of transcription units [24], and whole-genome screening of transcription regulatory elements [25, 26]. The PWM scores (PWMSs) for individual motifs have been found to be useful as a measure of the motif strength, for example, PWMS for individual splice sites has been used as a proxy of splicing efficiency in eukaryotes [27, 28]. 

PWM has been used not only as an independent tool for summarizing and predicting sequence motifs, but also as a key component in more advanced bioinformatic algorithms such as the variable-order Bayesian network [29], Gibbs sampler [30–32] and related algorithms based on the Monte Carlo method [33], MEME [34], and support vector machines [35–39]. While PWM has been used mainly to characterize and predict motifs in nucleotide sequences, recent studies have demonstrated its potential in characterizing and predicting functional protein motifs [40–42], signal peptides [43], and protein-protein-binding sites [44]. In particular, the method was successful in predicting tyrosine sulfation sites [45–48].

A PWM-based sequence analysis involves three types of output: the site-specific frequency distribution, the PWM itself, and PWMS for each input sequence (and optionally PWMS resulting from scanning new sequences with the trained PWM). Here I briefly review the PWM method in the context of motif discovery, followed by a detailed illustration of the Gibbs sampler of which PWM is a key component, and then propose statistical significance tests appropriate for each of the three types of PWM output. 

## 2. PWM in the Context of Motif Discovery Methods

The simplest input for a PWM-based method consists of an aligned set of sequences and the specification of the background (prior) frequencies. The main output of PWM, other than the PWM itself, consists of the site-specific information content and the motif information content [6] as well as PWMS for individual motifs, together with the associated statistical tests.

We first illustrate the PWM method by applying it to the 246 donor splice sites of yeast introns each represented by 5 nucleotide sites on the exon side and 12 nucleotide sites on the intron side (Table 1). The four columns on the left of Table 1 headed by A, C, G, and U are the site-specific counts of nucleotides A, C, G, and U. When all site-specific counts are greater than zero, each element in the PWM, designated by PWM_*ij*_ (where *i* = 1, 2, 3, and 4 corresponding to A, C, G, and U, respectively, and *j* is site index), is computed as

(1) $$\begin{array}{c} \text{PW}{\text{M}}_{ij}=\text{lo}{\text{g}}_{2}\left(\frac{p_{ij}}{p_{i}}\right), \end{array}$$

where *p*_*i*_ is the background frequency of nucleotide *i*, and *p*_*ij*_ is the site-specific nucleotide frequency for nucleotide *i* at site *j* (e.g., *p*_A1_ = 83/246 in Table 1). Plotting these site-specific PWM_*ij*_ values graphically over sites yields the sequence logo [49, 50]. The PWM score (PWMS) for a particular motif is computed as

(2) $$\begin{array}{c} \text{PWMS}=\sum_{j=1}^{L}\text{PW}{\text{M}}_{i,j}, \end{array}$$

where *L* is the length of the motif which equal 17 for our example shown in Table 1.

Note that PWMS is the logarithm of a likelihood ratio, or log-odds. Given a 17 mer, say, *S* = ACGGTACCACGTAAGTT, we have two hypotheses. The first hypothesis is that the 17 mer belongs to a motif, constrained to have specific nucleotides at specific sites (*θ*_Yes_), and the second is that each site in the 17 mer is sampled from a nucleotide pool with no site-specific constraints (*θ*_No_). The likelihoods of observing sequence *S*, given the two different hypotheses, are specified, respectively, as

(3) $$\begin{array}{c} L_{\text{Yes}}=p\left(S\;|\;\theta_{\text{Yes}}\right)=p_{\text{A}1}p_{\text{C}2}p_{\text{G}3}p_{\text{G}4}\cdots p_{\text{T}17},\\ L_{\text{No}}=p\left(S\;|\;\theta_{\text{No}}\right)=p_{\text{A}}^{5}p_{\text{C}}^{4}p_{\text{G}}^{4}p_{\text{T}}^{4}. \end{array}$$

This leads to the following that is identical to (2):

(4) PWMS=log2(LYesLNo)=log2pA1pA+log2pC2pC+⋯+log2pT17pT=PWMA1+PWMC2+⋯+PWMT17.

### 2.1. Specification of the Background Frequencies

The background frequencies (*p*_*i*_) have been specified in three different ways in previous publications. The first is simply to assume equal background frequencies [51, 52] in characterizing splice sites with PWM. This is equivalent to the classic sequence logo method for graphic display of site patterns [49] which does not take background frequencies into consideration. In sequences with biased nucleotide frequencies, equal *p*_*i*_ values will generate a false site pattern when there is in fact no pattern. For example, the AT-biased background genome in the yeast implies that PWM_A*j*_ and PWM_T*j*_ will be greater than PWM_C*j*_ and PWM_G*j*_ on average even when the sequences contain no site-specific information. Similarly, the classic sequence logo will display A and T more prominently than C and G even when the sequences of interest contains no site-specific information. 

The second approach to specify *p*_*i*_ is to compute it from the input sequences. As such, in our example, *p*_*i*_ can be computed from the four columns headed by A, C, G, and U on the left side of Table 1. This approach also has a problem. Suppose a certain motif is a poly-U sequence, and all input sequences are “UUUUUUUU”. This will generate background nucleotide frequencies with *p*_U_ = 1 and *p*_A_ = *p*_C_ = *p*_G_ = 0. Note that the site-specific frequencies, given the input sequences all being “UUUUUUUU”, are *p*_U*j*_ = 1 and *p*_A*j*_ = *p*_C*j*_ = *p*_G*j*_ = 0. So the resulting PWM would then suggest that the motif is not informative, which is contrary to our intuition, that is, a stretch of UUUUUUUU conserved across a set of aligned sequences is likely to be biologically informative. 

The third approach is to specify *p*_*i*_ according to the specific problem one wishes to solve. For example, when characterizing splice sites of introns in a particular species, one may use the nucleotide frequencies of all transcripts (including all exons and introns) annotated in the genome as the background frequencies [28]. Similarly, a study of site patterns of branchpoint sequences in introns could have *p*_*i*_ values computed from all intron sequences. I suggest that only this third approach be used to avoid the impression that PWM could have an infinite number of null hypotheses (each associated with a different specification of *p*_*i*_).

The computer program DAMBE [53, 54] offers different choices for specifying *p*_*i*_ in computing PWM. Similarly, the new sequence logo method allows more appropriate specification of background (prior) frequencies [50]. The resulting PWM for the 246 donor splice sites of yeast introns, with background frequencies computed from all introns, is shown in Table 1. 

### 2.2. Specification of Pseudocounts

When some *p*_*ij*_ values are zero, as is the case in our example, (1) is inapplicable because the logarithm of zero is undefined. Three approaches can be taken to avoid this problem [55, 56]. The first is to compute *p*_*ij*_ by

(5) $$\begin{array}{c} p_{ij}=\frac{f_{ij}+p_{i}}{N+1} \end{array}$$

which approaches *f*_*ij*_/*N* with increasing *N* (where *f*_*ij*_ is the site-specific count for nucleotide or amino acid *i* at site *j*). PWM_*ij*_ values can then be computed by (1). This approach is poor when *N* is small. 

The second approach is to use explicit pseudocounts by defining

(6) $$\begin{array}{c} f_{i\cdot \text{pseudo}}=\alpha f_{i},\\ f_{\text{pseudo}}=\sum_{i=1}^{M}f_{i\cdot \text{pseudo}}, \end{array}$$

where *f*_*i*_ is the frequency of nucleotide *i*, and *p*_*ij*_ is then

(7) $$\begin{array}{c} p_{ij}=\frac{f_{ij}+f_{i\cdot \text{pseudo}}}{N+f_{\text{pseudo}}}. \end{array}$$

It is important to keep *α* small (e.g., 0.0001) because the expected PWM_*ij*_ from random sequences is 0 in (1). A large *α* will substantially increase PWM_*ij*_ above 0 with random sequences. 

The two approaches above share one main disadvantage. Suppose we have 10 aligned motifs of 10 amino acids each. Position 3 is occupied by amino acids K (lysine) and R (arginine) and position 5 by amino acid E (glutamic acid). The two approaches above will specify pseudocounts for positions 3 and 5 in the same way, which is unreasonable for the following reason. If position 3 requires a positively charged amino acid, and position 5 a negatively charged amino acid, then amino acids K, R, and H (histidine) should be more likely found than other amino acids at position 3, and amino acid D (aspartic acid) should be more likely found than other amino acids at position 5. By using other aligned protein sequence data of roughly the same divergence we can derive frequency distributions for positions that require a positively charge or negatively charged amino acid and use these frequency distributions to produce pseudocounts [56]. In our case, the pseudocounts at positions 3 and 5 will be assigned quite differently because the frequency distribution for a position requiring a positively charged amino acid is typically quite different from that for a position requiring a negatively charged amino acid.

PWM and PWMS can potentially be used to measure codon usage bias. For example, given the frequency of nucleotide *i* as *p*_*i*_, the background frequency of a codon, say AGC, can be specified as *p*_A_ *p*_G_ *p*_C_, and compared to the observed frequency of AGC. Such an approach would eliminate one major weakness of commonly used codon bias indices such as CAI [57, 58] and Nc [59, 60].

## 3. Gibbs Sampler with PWM as a Key Component

**Figure 1:**
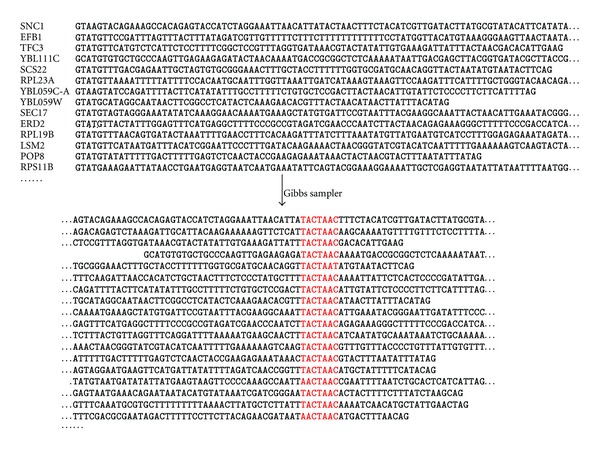
What Gibbs sampler does. The intron sequences in the top panel represent the input information to the Gibbs sampler. The bottom panel represents part of the output showing the identified motif (i.e., TAATAAC, in red) shared among the sequences. Output from DAMBE [53, 54]. The input intron sequence file (YeastAllIntron.fas) is in DAMBE installation directory in FASTA format.

While PWM is a technique for characterizing a set of identified motifs, Gibbs sampler [61], named after the mathematical physicist, J. W. Gibbs, is for *de novo* motif discovery. For example, given a set of yeast intron sequences, what and where is the branchpoint site? All information we have is that each intron should have one branchpoint site, but what sequence signature does it have and where is it located along the intron sequence? This scenario (Figure 1) is where the Gibbs sampler will shine. 

A similar scenario involves the discovery of regulatory equence motifs given a set of coexpressed genes (i.e., genes that increase or decrease their transcription level synchronously over time) by microarray [62, 63], SAGE [64, 65], or deep-sequencing [66–68] experiments. If the coexpressed genes are also coregulated, then they may share a certain yet-unknown transcription factor binding site controlled by the same or similar transcription factor. Given that the binding site is often located upstream of the translation initiation codon, one may extracted the upstream sequences from these coexpressed genes and let the Gibbs sampler to find the candidate regulatory motifs. A recent study has shown that shared motifs may also present in the 5′ UTR of mRNA to modulate translation initiation [69].

Gibbs sampler is one of the Monte Carlo algorithms that rely on repeated random sampling to estimate desired parameters. Monte Carlo method was envisioned by the famous mathematician Stanislaw Ulam, following the successful assembly of the first electronic computer ENIAC in 1945, and further developed by physicists and mathematicians working on nuclear weapon projects in the Los Alamos National Laboratory in mid-1940s [70]. The term “Monte Carlo method” was coined by Nicholas Metropolis to designate this class of computational algorithms. While the general application of the method unsurprisingly followed the operation of ENIAC in 1945, the physicist Enrico Fermi is known to have independently developed and applied the method nearly 15 years earlier with mechanical calculators [70].

Gibbs sampler simplifies computation in parameter estimation when analytical solution is very difficult or impossible to obtain. In biology, it has been used in the identification of functional motifs in proteins [31, 71, 72], biological image processing [73], pairwise sequence alignment [74], and multiple sequence alignment [75, 76]. However, the most frequent biological application of Gibbs sampler remains in the identification of regulatory sequences of genes [30, 77–84].

There are two slightly different applications of Gibbs sampler in motif prediction. The first assumes that each sequence contains exactly one motif [30] and the associated algorithm is called a site sampler. The second is more flexible and allows each sequence to have none or multiple motifs [71] and the algorithm is termed a motif sampler. We will illustrate the site sampler and then briefly discuss the motif sampler. 

I numerically illustrate the Gibbs sampler algorithm for motif discovery. The main output of the Gibbs sampler is typically of three parts. The first is the shared motif in an aligned format (bottom panel in Figure 1). The second is a PWM summarizing the discovered motif, and the third contains the associated significance tests which will be reviewed in a later section. The derived PWM, just like any other PWM, can be used to scan sequences not in the input data to discover the presence of the motif present elsewhere.

### 3.1. Computational Details of the Gibbs Sampler

**Figure 2:**
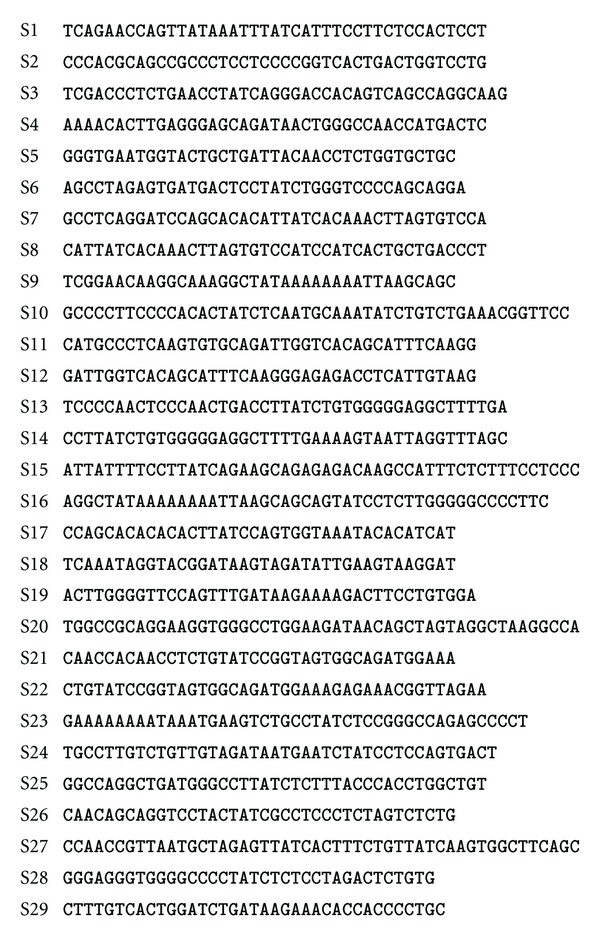
The erythroid sequences [85] for illustrating the Gibbs sampler algorithm, with the 3′-end trimmed to the maximum length 50 bases to fit the page.

We will use the erythroid nucleotide sequences [85], listed in Figure 2, to illustrate the Gibbs sampler algorithm. Our main objective is to infer the location and sequence of the unknown motif shared among the sequences so that we can align the motifs as shown in the bottom panel of Figure 1. The aligned motifs will allow us to generate a PWM that characterizes the motif by site-specific nucleotide frequency distributions. The PWM can be used to scan for the presence of the identified motif in other sequences. 

We need first to count all nucleotides, with their numbers designated as *F*_A_, *F*_C_, *F*_G_, and *F*_T_, respectively, in the sequences. The total number of nucleotides of all 29 sequences (Figure 2) is 1209, with *F*_A_, *F*_C_, *F*_G_, and *F*_T_ equal to 325, 316, 267, and 301, respectively. These values are needed for specifying pseudocounts (which we encountered in the previous section on PWM).

Let *N* be the number of input sequences designated as *S*_1_, *S*_2_,…, *S*_*i*_,…, *S*_*N*_. Let *L*_*i*_ be the length of *S*_*i*_, and *m* be the length of the motif, which typically is of length 4–8. For our illustration, we will use *m* = 6. One typically would run the Gibbs sampler several times with different *m* values if one knows little about the length of the motif. The PWM is of dimension 4 × *m* for nucleotide sequences, and 20 × *m* for amino acid sequences. Let A_*i*_ be the unknown starting position of the motif in *S*_*i*_. 

The main algorithm of Gibbs sampler is of two steps. The first is random initialization in which a random set of A_*i*_ values is assigned and site-specific nucleotide frequencies are calculated. The second step is predictive updating until a local solution of A_*i*_ values is obtained, together with site-specific nucleotide frequencies that can be made into a PWM. This is repeated multiple times and previously stored locally optimal solutions are replaced by better ones. Convergence is typically declared when two or more local solutions are identical. These steps are numerically illustrated in the following sections.

### 3.2. Initialization

The initiation step randomly assigns a value to A_*i*_, with the constraint that 1 ≤ A_*i*_ ≤ *L*_*i*_ − *m* + 1. So our first set of *N* “motifs” is essentially a random set of sequences of length m and is not expected to have any pattern. For readers who are curious, the first set of 29 random A_*i*_ values happen to be: 29, 31, 23, 28, 10, 2, 18, 32, 20, 15, 11, 25, 24, 30, 18, 15, 10, 23, 14, 15, 26, 36, 8, 6, 30, 19, 27, 26, and 14. The site-specific distribution of nucleotides from the 29 random motifs is shown in Table 2. There is hardly any site-specific pattern, as one would have expected. 

The second column in Table 2 will be referred to as the C0 vector with C0_A_, C0_C_, C0_G_, and C0_T_ equal to 278, 279, 230, and 248, respectively. The 4 × 6 matrix, occupying the last six columns in Table 2, will be referred to as the C matrix. The C matrix is tabulated from the 29 random motifs whereas the C0 vector is tabulated from nucleotides outside of the motifs. Thus, the sum of the first, second, third, and fourth rows of Table 2 should be equal to *F*_A_, *F*_C_, *F*_G_, and *F*_T_, respectively. Also note that each of the six columns in the C matrix should add up to 29.

### 3.3. Predictive Update

The predictive update consists of obtaining *N* (= 29 in our example) random numbers ranging from 1 to *N*, and use these numbers as an index to choose the sequences sequentially to update the site-specific distribution of nucleotides (the C matrix) and the associated frequencies (the C0 vector). For example, the *N* random numbers in my first run of the Gibbs sampler happen to be 11, 18, 26, 22, 2, 28, 12, 9, 7, 3, 17, 16, 1, 4, 21, 15, 14, 24, 19, 27, 29, 6, 10, 20, 13, 8, 23, 25, and 5, respectively. This means that *S*_11_ will be used first, and *S*_5_ last, for the first cycle of the predictive update. It is important to use a random series of numbers instead of choosing sequences according to the input order. The latter increases the likelihood of trapping Gibbs sampler within a local optimum.

Our first randomly chosen sequence happens to be *S*_11_ and its randomly chosen motif starts at site 11, that is, A_11_ = 11, with the motif being AGTGTG. This initial motif will now be taken out of the C matrix and put into the C0 vector. This motif has one A, zero C, three G's, and two U's. By adding these values to the C0 vector in Table 2, we obtain the C0 vector in Table 3. We also need to take this motif out of the C matrix by subtracting the first A from the first value in the first column in the C matrix in Table 2 (i.e., new C_A,1_ = old C_A,1_ − 1), the second G from the third value in the second column in the C matrix in Table 2 (i.e., new C_G,2_ = old C_G,2_ − 1), and so on. This converts the C matrix in Table 2 to the C matrix in Table 3. 

At this point the C matrix is made of the 28 randomly chosen motifs, one from each sequence (excluding *S*_11_). You will notice that each of the six columns in the C matrix has a sum of 28. The reason for taking the initial motif in *S*_11_ out of the C matrix and put it back into the C0 vector is that we are going to find a better motif in *S*_11_, and put it into the C matrix so that the C matrix will again be based on 29 motifs. How are we going to get a better motif? Recall that a position weight matrix (PWM) can be used to scan a sequence in a sliding window of length m to get position weight matrix scores (PWMSs) for each window. We will make a PWM out of the C0 vector and the C matrix and use the resulting PWM to scan *S*_11_ and get a new motif that has the highest PWMS.

One may wonder why such a practice would get us anywhere given the fact that the C matrix is initially made of random motifs. The resulting PWM would exhibit no pattern, and the resulting PWMSs will therefore be uninformative. The key concept here is that when one takes a random walk over a terrain with multiple peaks, one sooner or later will encounter a peak, and climbing the peak will at least bring us to a local maximum. After reaching the top of one peak and recording the height, we will land ourselves at another randomly chosen location and start climbing local peaks again. This process continues until we reach the highest peak or after a fixed number of computer iterations without finding any higher peak.

Typically, the PWM is generated by using the C0 vector as background frequencies (*p*_*i*_) and the C matrix as site-specific frequencies *p*_*ij*_. However, although most algorithmic illustration of the Gibbs sampler computes *p*_*i*_ this way (e.g., [32, pp. 133–147]), *p*_*i*_ computed from the C0 vector has serious problems when input sequences are almost as short as the motif. For example, if the true motif has many nucleotide A and few nucleotide U, then the C0 vector will also have many A and few U. Now a motif with a few nucleotide U will be taken as deviating substantially from the background and will tend to have a high PWMS, leading to a biased estimate of the true motif. Thus, when input sequences are short, one should specify the background frequencies instead of using C0 to compute *p*_*i*_. One may refer to the previous section on PWM for more information on background frequencies.

For pseudocounts, we may use *α* = 0.0001. The resulting PWM is then used to scan *S*_11_ which is 40 bases long, with 35 ( = 40 − *m* + 1) possible motif starting points (i.e., possible A_*i*_ values along the sequence). The 35 PWMS values for these 35 possible motifs in *S*_11_ (Table 4) are normalized to have a sum of 1 (*P*_Norm_ in Table 4). We now proceed to update the initial A_11_ ( = 11) by a new A_11_ value based on result in Table 4. How should we choose the new A_11_ value? 

There are two strategies to choose the new A_11_ value. The first is to randomly pick up an A_*i*_ value according to the magnitude of *P*_Norm_ (Table 4). You may visualize a dartboard with 35 slices with their respective areas being proportional to *P*_Norm_ values. When you throw a dart at the dartboard, large slices will have a better chance of being hit than small slices. If the dart happens to land on the 7th slice, then the initial A_11_ = 11 will be updated to A_11_ = 7, with the original motif AGTGTG replaced by the new motif CTCAAG.

The second strategy is simply to use the largest *P*_Norm_ value for updating initial A_11_ to the new A_11_ value. As the motif starting at site 25 has the largest *P*_Norm_, we will set the new A_11_ equal to 25 and replace the initial motif (= AGTGTG) by the new motif (= TCACAG). With this approach we do not need *P*_norm_ as we can choose A_11_ based on the largest odds ratio in Table 4. This strategy is faster than the first, but did not seem to lose any sensitivity in motif discovery based on limited simulation studies. However, if one is concerned about the possibility of missing motifs, one should use the first strategy.

Regardless of how the new A_11_ is chosen, the updating is the same. Suppose we have taken the second strategy and set the new A_11_ equal to 25. The C matrix in Table 2 is then revised by replacing the original A_11_ motif (= AGTGTG) by the new motif (= TCACAG). This leads to an updated C0 vector and C matrix (Table 5).

We repeat this process for the rest of the sequences to update the rest of A_*i*_ values. After the last sequence has been updated, we have obtained a new set of A_*i*_ values, a new set of 29 motifs, together with the PWM based on the associated C0 vector and C matrix. At this point we compute a weighted alignment score (i.e., a weighted PWMS) as follows:

(8) $$\begin{array}{c} F=\sum_{i=1}^{N_{\text{Code}}}\sum_{j=1}^{m}C_{i,j}\text{PW}{\text{M}}_{ij}, \end{array}$$

where *m* is the motif width, and *N*_Code_ is the number of different symbols in the sequences (4 for nucleotide and 20 for amino acid sequences). *F* is a measure of the quality of alignment of the motifs. The larger the *F* value, the better. 

The *F* value, as defined in (8), has many different names. It has been called the Kullback-Leibler information or Kullback-Leibler divergence in information theory [86–88], or large-deviation rate function in statistical estimation [89]. In bioinformatics, especially in motif characterization and prediction involving a PWM, it is most often referred to as the information content [6]. The fact that the Kullback-Leibler information is a special case of the so-called *f*-divergence that measures the difference between two probability distributions *P* and *Q* leads naturally to the use of the letter *F* in (8). 

The predictive updating is repeated again and again. Each time when we get a new set of A_*i*_ values, a new set of motifs and the PWM based on the C0 vector and the C matrix, we compute a new *F* value. If the new *F* value is greater than the previously stored *F* value, then the new *F* value, the new set A_*i*_ values, and the new set of motifs will replace the previously stored ones. This continues until we reach a local maximum of *F* or when the preset maximum number of local loops has been reached. The resulting *F* value, the set of A_*i*_ values, the new set of motifs and the associated PWM are stored as the locally optimal output. In the hill-climbing analogy, *F* represents the height of a local peak.

 The entire process is now repeated from the very beginning, that is, we again perform the initialization by choosing another random set of A_*i*_ values, and go through the local iteration to obtain another locally optimal output. If the new locally optimal output is better than previously stored ones (i.e., the new *F* value is larger than the previously stored one), the new output will replace the previously stored output. This process is repeated multiple times until convergence is reached, that is, when new *F* values are consistently the same as the previously stored one, or until a fixed number of computation iteration has been reached without finding an *F* value better than what has already been recorded. The final site-specific nucleotide distribution (Table 6) displays a much stronger pattern than the initial distribution (Table 2) from 29 randomly chosen motifs. 

The final aligned motifs (Figure  7-2 in [32]) share in general a consensus of (C/T)TATC(A/T). Its reverse complement (A/T)GATA(A/G) is known to be the binding site of GATA-binding transcription factors [90–95]. This discovery of the motif suggests that this set of sequences may indeed be coregulated by the same type of GATA-binding transcription factors. Such findings are crucial in transcriptomic and proteomic studies aiming to understand gene regulation networks. Algorithms such as Gibbs sampler help us understand interactions among genes and gene products.

It might be relevant here to summarize essential biology about the GATA box and GATA-binding transcription factors. A living cell is a system with many genetic switches that can be turned on or off in response to intracellular and extracellular environment. It is these switches that distinguish a normal living cell from a cancer cell or a dead cell. The GATA motif (or GATA box) is one of such switches and it is switched on or off by specific transcription factors (which are proteins that bind to the motif and turn on or off the transcription of the gene containing such motifs). One of the better known GATA-binding transcription factors is GATA-1 which binds to the GATA motif found in cis-elements of the vast majority of erythroid-expressed genes of all vertebrate species examined [96, 97]. The core promoter of the rat platelet factor 4 (PF4) gene contains such a GATA motif and the binding of such GATA motif by GATA-binding proteins such as GATA-1 suppresses the transcription of the PF4 gene [91]. It is now known that GATA regulatory motifs and the GATA-binding transcription factors are present in a variety of organisms ranging from cellular slime mold to vertebrates, including plants, fungi, nematodes, insects, and echinoderms [98], suggesting that the function of the genetic switch is far beyond erythropoiesis. In human, the GATA motif and the GATA-binding proteins are implicated in several diseases [99]. The sequence divergence of GATA motifs and their binding proteins should shed light on the coevolution of the components of genetic switches.

One may have noted that some sequences have a strong (C/T)TATC(A/T) motif, whereas others (e.g., the second, the fourth and the fifth sequences) have only weak and highly doubtful signals. Computer programs implementing Gibbs sampler typically would output a quantitative measure of the strength of the signal, and PWMS is the most often used index for this purpose (Table 7). Recall that PWMS is the log-odds, but one may use the odds ratio directly as a measure of relative motif strength. Also recall that an odds ratio is the ratio of two probabilities associated with two hypotheses. Define *θ*_Yes_ as the hypothesis that the 6-mer is a motif with its site-specific constraints, and *θ*_No_ as the hypothesis that the 6-mer is not a motif and has its probabilities specified only by the four overall nucleotide frequencies. The odds ratio is the ratio of the probability that *θ*_Yes_ is true over the probability that *θ*_No_ is true. One generally should take a cut-off value of 20, that is, *θ*_Yes_ is 20 times more likely than *θ*_No_. 

One should note that Gibbs sampler, being started from a random set of A_*i*_ values, may not necessarily converge to the same motif. This is both an advantage and a disadvantage of the algorithm. The advantage is that repeated running of the algorithm will allow us to identify other types of hidden motifs (i.e., other than the reverse complement of the GATA motif) in the sequences. The disadvantage is that users not familiar with the algorithm often get confused when the same input generates quite different results. For example, another set of putative motifs, in the form of RGVAGR (where R is A or G and V is “not T”), has been found to be shared among the sequences [32, p. 146].

It is possible that the input sequences may contain two or more different biologically significant motifs. If one motif is much stronger (more over-represented among the input sequences) than other motifs, and if the search by the Gibbs sampler algorithm outlined before is exhaustive, then we will always end up with the strongest motif and miss all other biologically interesting motifs. However, one could run Gibbs sampler by specifically exclude the strongest motif already identified so that weaker motifs can then be identified.

### 3.4. Motif Sampler

The Gibbs sampler has two versions. The one that we have just illustrated is called site sampler. It assumes that each sequence contains exactly one motif [30]. The other version is more flexible and allows each sequence to have none or multiple motifs [71] and the algorithm is termed motif sampler. The GATA-binding transcription factors comprise a protein family whose members contain either one or two highly conserved zinc finger DNA-binding domains [98] and it is consequently likely that a sequence may contain more than one GATA box. For example, the erythroid Kruppel-like factor (EKLF, which is a zinc finger transcription factor required for *β*-globin gene expression) has in its 5′-region two GATA motifs flanking an E box motif characterized by CANNTG [100]. This calls for an algorithm that can identify multiple motifs in a single sequence.

The site sampler can be extended to motif sampler by post-processing. The PWM generated from the site sampler can be used to re-scan the sequences for motifs and compute the associated PWMS or odds ratio for all 6-mers in each sequence. All what we need is to have a cut-off score to keep those motifs with a PWMS or odds ratio greater than the cut-off score. 

## 4. Statistical Significance Tests

The PMW, be it from alignment of known motifs or from running the Gibbs sampler, need to be assessed for its statistical significance. One continuous problem with PWM is the lack of generally applicable and accurate significance tests, either for individual sites of the motif, on PWM or on PWMS. There are two reasons why accurate significance tests are desirable. First, after characterizing a motif with PWM, one naturally wants to know whether the characterized PWM is significant, which sites contribute to the significance and which sequence has a PWMS that is significantly greater than random expectation. Second, after finding a significant PWM, one typically would want to use the PWM to scan other sequences to identify new motifs, and one needs a good significance test to show the reliability of the identified motif. This would reduce the number of putative sequence motifs going through experimental verification which is typically tedious and expensive [101, 102]. 

In short, three separates significance tests are required: one for individual sites, one for PWM per se and one for PWMS. These tests are detailed in the following sections.

### 4.1. Statistical Significance Tests for Individual Sites

The statistical significance of individual sites can be done by *χ* ^2^-tests with type I error rate controlled for by the false discovery rate [103, 104]. Take the data in Table 1 for example. The background frequencies are A = 0.3279, C = 0.1915, G = 0.2043, and U = 0.2763, which allow us to obtain expected counts of A, C, G, and T. With 17 *χ* ^2^-tests (Table 1), we face the problem of multiple comparisons and need to control for the familywise error rate [105] which is synonymous to experimentwise error rate.

Designate the error rate by *α*_0_, then the exact critical *α* for rejection in individual tests is

(9) $$\begin{array}{c} \alpha =1-\left[{\left(1-\alpha_{0}\right)}^{1/N}\right], \end{array}$$

where *N* is the number of tests and is equal to 17 in our case. If we set *α*_0_ = 0.05, then *α* = 0.003012705. The Bonferroni criterion is based on the approximation that

(10) $$\begin{array}{c} \alpha =1-\left[{\left(1-\alpha_{0}\right)}^{1/N}\right]\;\approx \frac{\alpha_{0}}{N}, \end{array}$$

which leads to *α* = 0.002941176. The second order Bonferroni *α*, when relevant assumptions are met [105], is based on

(11) $$\begin{array}{c} N\alpha -\left(N-1\right)\alpha^{2}=\alpha_{0}, \end{array}$$

which leads to *α* = 0.0029493634. In practice, these different *α* values make little difference. In our case, all three *α* values lead to the conclusion that the frequency distribution at sites 1, 2, 13, and 16 do not deviate significantly from the background frequencies.

The statistical protocol for controlling for the familywise error rate has been considered too conservative, and the protocol for controlling for the false discovery rate (FDR) has consequently been proposed recently [103, 104]. The classical FDR approach [103], now commonly referred to as the Benjamini-Hochberg procedure or simply the BH procedure, sorts *p* values in descending order and computes *p*_critical·BH·*i*_ for the *i*th *p* value (where the subscript BH stands for the BH procedure) as

(12) $$\begin{array}{c} p_{\text{critical}\cdot \text{BH}\cdot i}=\frac{q\cdot i}{N}, \end{array}$$

where *q* is FDR (e.g., 0.05), *i* is the rank of the *p* value in the sorted array of *p* values, and *N* is the number of tests (i.e., the number of *p* values). If *k* is the largest *i* satisfying the condition of *p*_*i*_ ≤ *p*_critical·BH·*i*_, then we reject hypotheses from *H*_1_ to *H*_*k*_. In our case, all the sites are statistically significant based on *p*_critical·BH·*i*_ (Table 8).

The FDR procedure above assumes that the test statistics are independent or positively dependent (in the extreme case of perfect positive dependence, all tests are the same and there is really only just one test with no multiple comparison problem). A more conservative FDR procedure has been developed that relaxes the assumption [104]. This method, now commonly referred to as the Benjamini-Yekutieli or simply the BY procedure, computes *p*_critical·BY·*i*_ for the *i*th hypothesis as

(13) $$\begin{array}{c} p_{\text{critical}\cdot \text{BY}\cdot i}=\frac{q\cdot i}{N\sum_{i=1}^{N}\left(1/i\right)}=\frac{p_{\text{critical}\cdot \text{BH}\cdot i}}{\sum_{i=1}^{N}\left(1/i\right)}. \end{array}$$

With *N* = 17 in our case, Σ1/*k* = 3.439552523. Based on *p*_critical·BY·*i*_, the *χ* ^2^-tests pertaining to sites 1 and 2 are not statistically significant (Table 8). The BY procedure was found to be too conservative and several alternatives have been proposed [106]. For large *N*, Σ1/*k* converges to ln⁡(*N*) + *γ* (Euler's constant equal approximately to 0.57721566). Thus, for *N* = 10000, Σ1/*k* is close to 10. So *p*_critical·BY_ is nearly 10 times smaller than *p*_critical·BH_. Both FDR procedures above have been used in significance tests concerning yeast splicing sites [23].

### 4.2. Evaluating Statistical Significance of PWM When Pseudocounts Are Used

Whether a PWM represents a motif with site-specific constraints can be tested by using the *F* statistic [6] specified in (8). However, the distribution of *F* is altered by pseudocounts as specified in (5) and (7). For example, the expectation of *F* is no longer zero with pseudocounts when there is no site-specific pattern. 

A more straightforward method for evaluating the significance of PWM is by resampling. With the tetranomial distribution defined by (*p*_A_+*p*_C_+*p*_G_+*p*_T_)^*N*^, where *p*_*i*_ is the nucleotide frequency of nucleotide *i*, we can obtain a new set of sequences (246 sequences of 17 nt each) and compute *F*. This is repeated for, say, 5000 times to obtain 5000 *F* values. The 95th or 99th percentile of the *F* values can be taken as critical *F* values at 0.05 and 0.01 significance levels, respectively. An observed *F* for the PWM is significant if it is greater than the critical *F*. Based on this criterion, the PWM from the 246 donor splice sites is highly significant. The same resampling technique can also be used to evaluate the significance of the site-specific patterns in the previous section or the significance of PWMS in the next section.

### 4.3. Statistical Significance of PWMS

One of the purposes of constructing a PWM is to facilitate the computation of PWMSs. For example, the PWMS for sequence UAAAGGUAUGUUUAAUU, given the PWM in Table 1 (the four columns headed by A, C, G, and U on the right side), is simply

(14) $$\begin{array}{c} \text{PWMS}=\text{PW}{\text{M}}_{\text{U}1}+\text{PW}{\text{M}}_{\text{A}2}+\cdots +\text{PW}{\text{M}}_{\text{U}17}. \end{array}$$

Thus, we can use the PWM to predict a new donor splice site by scanning a nucleotide sequence with a window of 17 nucleotide sites and computing the PWMS. The larger the PWMS, the more likely the 17-mer is a donor splice site. However, we need to address the question of how large is large in such *in silico* predictions.

**Figure 3:**
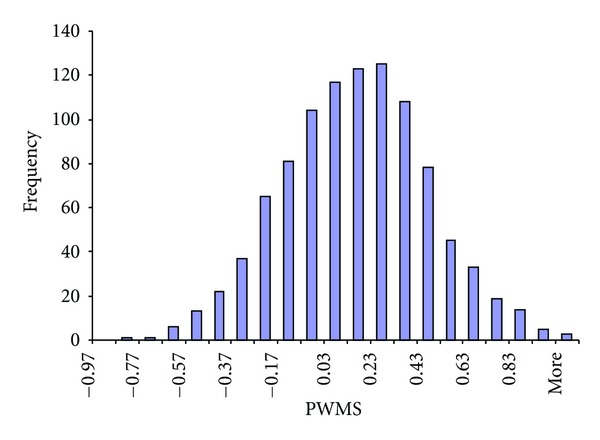
PWMS from random sequences follows approximately the normal distribution, based on 1000 random sequences of length 17 drawn from the pool of nucleotides with frequencies of A, C, G, and T equal to 0.3279, 0.1915, 0.2043, and 0.2763, respectively. The distribution has mean equal to 0.068884 and standard deviation equal to 0.314714254.

PWMS from random sequences follows approximately the normal distribution (Figure 3), with mean 0 (or slightly greater than 0 when pseudocounts are used with a small *α*). The distribution in Figure 3 has a mean equal to 0.068884 and a standard deviation equal to 0.314714254. 

Suppose we are to use our 4 × 17 PWM to scan a target sequence *S* of 1000 nt for a possible donor splice site. There are 984 ( = 1000 − 17 + 1) different 17 mers along the sequence *S*, resulting in 984 PWMS values. If the maximum PWMS is 1, how statistically significant is it? 

If the length of the target sequence *S* were only 17 nt instead of 1000 nt, then the answer is easy. The upper 99% confidence limit for a normal distribution with mean equal to 0.0689 and standard deviation equal to 0.3147 is 0.8808 ( = 0.06888 + 2.58 × 0.3147), which implies that a PWMS of 1 is significant at the 0.01 level. However, because our target sequence *S* is 1000 nt, with the maximum of PWMS equal to 1 out of a total of 984 PWMS values, we need to go a long way to evaluate the significance of this maximum PWMS value.

Suppose we perform many sampling experiments from the same normal distribution *p*(*x*) as in Figure 3:

(15) $$\begin{array}{c} p\left(x\right)=\frac{e^{{\left(x-\mu \right)}^{2}/(2\sigma^{2})}}{\sigma \sqrt{2\pi }}. \end{array}$$

In each experiment, we sample *N* times to obtain *x*_1_, *x*_2_,…, *x*_*N*_. The maximum *x* in each experiment is *x*_max⁡_. This is equivalent to use PWM to scan a sequence to obtain PWMS_1_, PWMS_2_,…, PWMS_N_, with the maximum PWMS designated as PWMS_max⁡_. What is the distribution of *x*_max⁡_, designated as *F*(*x*_max⁡_)? Note that *x*_max⁡_ is an extreme value of *N* *x* values, so it is natural to call *F*(*x*_max⁡_) an extreme value distribution (EVD). 

Extreme value distribution or EVD, also referred to as the Gumbel distribution in honour of the pioneer of the statistics of extremes [107], is used in BLAST [108, 109] and new versions of FASTA [110] to attach statistical significance to a match score between two sequences. It is also used to perform significance tests involving PWM [5, 6, 55]. Here I will outline the mathematical framework of EVD pertaining to PWMS. 

The probability of getting an *x* value smaller than *x*_max⁡_ is

(16) $$\begin{array}{c} G\left(x<x_{\max}\right)=\int_{0}^{x_{\max}}p\left(x\right)dx. \end{array}$$

**Figure 4:**
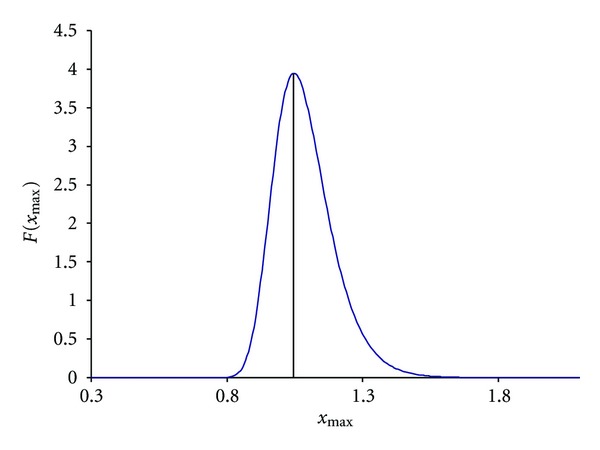
Extreme value distribution as specified in (17), with *μ* = 0.068884, *σ* = 0.314714254, and *N* = 984.

Note that *x*_max⁡_ can be either *x*_1_, *x*_2_,…, or *x*_*N*_, with *N* possibilities. (*N* − 1)  *x*_*i*_ values are smaller than *x*_max⁡_ in each experiment. This leads us to

(17) $$\begin{array}{c} F\left(x_{\max}\right)=Np\left(x_{\max}\right)G{\left(x<x_{\max}\right)}^{N-1}, \end{array}$$

which is plotted for *μ* = 0.068884, *σ* = 0.314714254, and *N* = 984 (Figure 4). Compared to the distribution of *p*(*x*) in Figure 3, the distribution of *F*(*x*_max⁡_) has been shifted substantially to the right and peaks at *x*_max⁡_ = 1.05. 

Now we can answer the question of whether our observed *x*_max⁡_ = 1 is statistically significant. The probability of observing an *x*_max⁡_ value equal to 1 or greater is

(18) $$\begin{array}{c} p\left(x_{\max}\geq x_{\text{obs}}\right)=\int_{x_{\text{obx}}}^{\infty }F\left(x_{\max}\right)dx_{\max}, \end{array}$$

which is approximately 0.7986, that is, it is not statistically significant.

A much simpler, but likely less accurate, method based on *p*(*x*) only without deriving (16)–(18), is to use the Bonferroni criterion in (10). With *α*0 = 0.05, *α* = *α*_0_/986 = 0.00005081 which requires a PWMS value equal to 1.292076814 to be marginally significant, given *μ* = 0.068884, and *σ* = 0.314714254. As our observed maximum PWMS is 1 < 1.292076814, it is not significant at the 0.05 significance level.

In summary, a PWM-based sequence analysis involves three types of output: the site-specific deviation from the background frequencies, the position weight matrix itself and the position weight matrix score for each input sequence. The significance of the first can be evaluated with *χ* ^2^-tests using the false discovery rate as the criterion for rejection of the null hypothesis, the second by the resampling method, and the third by statistics based extreme value distribution. These tests have been implemented in the most recent versions of DAMBE [53, 54].

## Figures and Tables

**Table 1:** Site-specific frequencies and position weight matrix (PWM) for 246 donor splice sites (each represented by 5 sites on the exon side and 12 sites on the intron side). The *χ* ^2^ test is performed for each site against the expected background frequencies with A = 0.3279, C = 0.1915, G = 0.2043, and U = 0.2763. Sites that have been experimentally verified to be important are in bold.

Site	A	C	G	U	*χ* ^ 2^	P	A	C	G	U
1	83	30	49	84	10.10	0.0177	0.0525	−0.6332	−0.0260	0.3143
2	103	44	46	53	10.04	0.0182	0.3613	−0.0878	−0.1162	−0.3434
3	121	36	38	51	30.01	0.0000	0.5920	−0.3739	−0.3886	−0.3981
4	122	38	33	53	32.16	0.0000	0.6038	−0.2969	−0.5893	−0.3434
5	81	40	81	44	28.33	0.0000	0.0177	−0.2238	0.6933	−0.6081
6	0	1	245	0	948.34	0.0000	−6.6464	−5.0056	** 2.2841 **	−6.6469
7	0	9	0	237	582.23	0.0000	−6.6464	−2.3190	−6.6480	**1.8032**
8	239	1	2	4	462.46	0.0000	** 1.5693 **	−5.0056	−4.3320	−3.8633
9	16	24	1	205	387.81	0.0000	−2.2655	−0.9496	−5.0680	** 1.5946 **
10	2	0	243	1	928.96	0.0000	−4.8476	−6.6483	** 2.2723 **	−5.3416
11	9	7	2	228	521.06	0.0000	−3.0427	−2.6612	−4.3320	** 1.7475 **
12	87	15	34	110	53.66	0.0000	0.1198	−1.6111	−0.5468	0.7006
13	84	49	30	83	11.71	0.0085	0.0696	0.0659	−0.7246	0.2971
14	111	39	33	63	19.09	0.0003	0.4684	−0.2599	−0.5893	−0.0969
15	106	38	31	71	17.24	0.0006	0.4024	−0.2969	−0.6781	0.0738
16	92	30	40	84	13.69	0.0034	0.1997	−0.6332	−0.3155	0.3143
17	80	38	36	92	14.32	0.0025	−0.0001	−0.2969	−0.4655	0.4445

**Table 2:** Site-specific distribution of nucleotides from the 29 random motifs of length 6. The second column lists the distribution of nucleotides outside the 29 random motifs.

		Site					
Nuc	C0	1	2	3	4	5	6

A	278	8	7	9	6	10	7
C	279	3	8	5	10	6	5
G	230	7	5	6	5	3	11
T	248	11	9	9	8	10	6

**Table 3:** Site-specific distribution of nucleotides from the 28 random motifs of length 6, after removing the initial motif in *S*_11_. The second column lists the distribution of nucleotides outside the 28 random motifs.

		Site					
Nuc	C0	1	2	3	4	5	6

A	279	7	7	9	6	10	7
C	279	3	8	5	10	6	5
G	233	7	4	6	4	3	10
T	250	11	9	8	8	9	6

**Table 4:** Possible locations of the 6-mer motif along *S*_11_, together with the corresponding motifs and their position weight matrix scores expressed as odds ratios. The last column lists the odds ratios normalized to have a sum of 1.

Site	6-mer	Odds Ratio	P_Norm_
1	CATGCC	0.153	0.004
2	ATGCCC	0.850	0.021
3	TGCCCT	0.664	0.016
4	GCCCTC	0.944	0.023
5	CCCTCA	0.254	0.006
6	CCTCAA	0.843	0.021
7	CTCAAG	0.609	0.015
8	TCAAGT	0.717	0.018
9	CAAGTG	0.613	0.015
10	AAGTGT	0.426	0.011
11	AGTGTG	0.967	0.024
12	GTGTGC	0.546	0.014
13	TGTGCA	0.594	0.015
14	GTGCAG	4.034	0.100
15	TGCAGA	0.251	0.006
16	GCAGAT	1.084	0.027
17	CAGATT	0.343	0.009
18	AGATTG	1.812	0.045
19	GATTGG	1.128	0.028
20	ATTGGT	0.408	0.010
21	TTGGTC	1.194	0.030
22	TGGTCA	0.888	0.022
23	GGTCAC	1.005	0.025
24	GTCACA	0.596	0.015
25	TCACAG	5.888	0.146
26	CACAGC	0.064	0.002
27	ACAGCA	0.569	0.014
28	CAGCAT	0.569	0.014
29	AGCATT	0.381	0.009
30	GCATTT	2.024	0.050
31	CATTTC	0.474	0.012
32	ATTTCA	1.317	0.033
33	TTTCAA	4.293	0.107
34	TTCAAG	2.475	0.061
35	TCAAGG	1.279	0.032

**Table 5:** Site-specific distribution of nucleotides from the 29 initial motifs of length 6, after replacing the initial A_11_ motif (= AGTGTG) by the new motif (= TCACAG).

		Site					
Nuc	C0	1	2	3	4	5	6

A	277	7	7	10	6	11	7
C	277	3	9	5	11	6	5
G	232	7	4	6	4	3	11
T	249	12	9	8	8	9	6

**Table 6:** Final site-specific distribution of nucleotides from the 29 identified motifs. Output from DAMBE [53, 54].

		Site					
Nuc	C0	1	2	3	4	5	6

A	275	3	0	22	0	9	16
C	285	11	0	0	0	19	1
G	252	0	7	7	0	0	1
T	223	15	22	0	29	1	11

**Table 7:** Output of PWMS as a quantitative measure of the strength of the identified motifs. Output from DAMBE [53, 54].

SeqName	Motif	Start	Odds-ratio
Seq1	TTATCA	18	163.6602
Seq2	CGGTCA	22	14.5511
Seq3	CTATCA	14	101.8203
Seq4	AGATAA	17	9.1127
Seq5	TGATTA	16	12.9266
Seq6	CTATCT	18	90.7790
Seq7	TTATCA	20	163.6602
Seq8	TTATCA	2	163.6602
Seq9	CTATAA	17	58.1420
Seq10	CTATCT	14	90.7790
Seq11	TGGTCA	21	23.3886
Seq12	TTGTAA	33	38.9024
Seq13	TTATCT	20	145.9129
Seq14	TTATCT	2	145.9129
Seq15	TTATCA	10	163.6602
Seq16	CTATAA	3	58.1420
Seq17	TTATCC	13	34.3258
Seq18	AGATAT	20	8.1245
Seq19	TGATAA	16	32.0835
Seq20	AGATAA	24	9.1127
Seq21	CTGTAT	12	21.5783
Seq22	CTGTAT	0	21.5783
Seq23	CTATCT	23	90.7790
Seq24	TTGTCT	4	60.7395
Seq25	TTATCT	17	145.9129
Seq26	CTATCG	15	21.2368
Seq27	TTATCA	19	163.6602
Seq28	CTATCT	15	90.7790
Seq29	TTGTCA	2	68.1272

Mean			76.3120
Stdev			57.8163

**Table 8:** Evaluating statistical significance of individual sites by two types of false discovery rate.

Site	*p*	pBH^(1)^	pBY^(2)^
6	*0.0000000000^†^	0.002941	0.000855
10	*0.0000000000^†^	0.005882	0.001710
7	*0.0000000000^†^	0.008824	0.002565
11	*0.0000000000^†^	0.011765	0.003420
8	*0.0000000000^†^	0.014706	0.004276
9	*0.0000000000^†^	0.017647	0.005131
12	*0.0000000000^†^	0.020588	0.005986
4	*0.0000004842^†^	0.023529	0.006841
3	*0.0000013734^†^	0.026471	0.007696
5	*0.0000030965^†^	0.029412	0.008551
14	*0.0002619304^†^	0.032353	0.009406
15	*0.0006307900^†^	0.035294	0.010261
17	*0.0025004071^†^	0.038235	0.011116
16	*0.0033589734^†^	0.041176	0.011971
13	*0.0084455695^†^	0.044118	0.012827
1	*0.0177349476	0.047059	0.013682
2	*0.0182291629	0.050000	0.014537

^(1)^Critical *p* based on Benjamini and Hochberg (1995) [103].

^(2)^Critical *p* based on Benjamini and Yekutieli (2001) [104].

*Significant by the criterion in Benjamini and Hochberg (1995) [103].

^†^Significant by the criterion in Benjamini and Yekutieli (2001) [104].
